# Providers' perspectives on the vaginal birth after cesarean guidelines in Florida, United States: a qualitative study

**DOI:** 10.1186/1471-2393-11-72

**Published:** 2011-10-12

**Authors:** Kim J Cox

**Affiliations:** 1 College of Nursing, University of New Mexico, MSC 09 5350, 1 University of New Mexico, Albuquerque, NM, 87131-0001, USA

## Abstract

**Background:**

Women's access to vaginal birth after cesarean (VBAC) in the United States has declined steadily since the mid-1990s, with a current rate of 8.2%. In the State of Florida, less than 1% of women with a previous cesarean deliver vaginally. This downturn is thought to be largely related to the American College of Obstetricians and Gynecologists (ACOG) VBAC guidelines, which mandate that a physician and anesthesiologist be "immediately available" during a trial of labor. The aim of this exploratory qualitative study was to explore the barriers associated with the ACOG VBAC guidelines, as well as the strategies that obstetricians and midwives use to minimize their legal risks when offering a trial of labor after cesarean.

**Methods:**

Semi-structured interviews were conducted with 11 obstetricians, 12 midwives, and a hospital administrator (*n *= 24). Interviews were recorded and transcribed verbatim, and thematic analysis informed the findings.

**Results:**

Fear of liability was a central reason for obstetricians and midwives to avoid attending VBACs. Providers who continued to offer a trial of labor attempted to minimize their legal risks by being highly selective in choosing potential candidates. Definitions of "immediately available" varied widely among hospitals, and providers in solo or small practices often favored the convenience of a repeat cesarean delivery rather than having to remain in-house during a trial of labor. Midwives were often marginalized due to restrictive hospital policies and by their consulting physicians, even though women with previous cesareans were actively seeking their care.

**Conclusions:**

The current ACOG VBAC guidelines limit US obstetricians' and midwives' ability to provide care for women with a previous cesarean, particularly in community and rural hospitals. Although ACOG has proposed that women be allowed to accept "higher levels of risk" in order to be able to attempt a trial of labor in some settings, access to VBAC is unlikely to increase in Florida as long as systemic barriers and liability risks remain high.

## Background

In the United States and other developed countries, the vaginal birth after cesarean (VBAC) rate has been steadily declining for more than a decade [1,2]. Only 8.2% of US women with a previous cesarean delivery attempted a vaginal birth in 2007, compared with 35.3% in 1997, despite evidence that 60% to 80% of VBACs are successful [1]. A similar trend is apparent in Australia, where VBAC rates declined from 31% in 1998 to 19% in 2006 [2]. Although VBAC has been extensively validated as a safe option for most women with a previous cesarean [1], non-medical factors are thought to be driving the decline in rates since medical factors have changed little over the years [3].

Prior to the 1970s, the phrase "once a cesarean, always a cesarean" [[4], p. 1] dominated obstetrical practice in the United States and throughout much of the world. As surgical techniques became safer in the 1970s, however, cesarean rates began to rise [3]. Concerns over the rising cesarean rate in the United States prompted the National Institutes of Health (NIH) to convene the first Consensus Development Conference Panel on Cesarean Childbirth in 1981. The panel recommended that a trial of labor was a safe and reasonable alternative to an elective repeat cesarean delivery for carefully selected women [5]. During the 1980s and early 1990s, results from a number of large, multicenter prospective trials provided evidence to support the safety of VBAC [6,7]. As a result, VBAC rates rose from 6.6% in 1985 to 28.3% in 1996 [8]. The American College of Obstetricians and Gynecologists (ACOG) also published a series of VBAC guidelines that were successively less restrictive and suggested that a trial of labor be encouraged in women who were at low risk for complications [9,10]. Interest by women and their providers resulted in an increased number of successful VBACs. Moreover, in the interest of cost savings, some institutions and insurers even required women to undergo a trial of labor [11].

The downward trend in VBAC began in 1996, following the publication of an article by McMahon and colleagues in the *New England Journal of Medicine *[12]. The McMahon study, which was conducted in Nova Scotia, Canada, reported that major obstetrical complications were nearly twice as likely in the trial of labor group as in those women who underwent an elective repeat cesarean [12]. The concern that was generated by this study, along with increasing liability pressure on obstetricians, prompted ACOG to publish a revised set of guidelines in 1999. Although ACOG concluded that most women should continue to be counselled about VBAC and offered a trial of labor, the language regarding physician availability was changed from "readily available" to "immediately available throughout active labor" [[9], p. 198]. The ACOG guidelines also specified that "VBAC should be attempted in institutions equipped to respond to emergencies with physicians immediately available to provide emergency care" [p. 201]; this recommendation, however, was based primarily on consensus and expert opinion, rather than on research evidence [9].

**Figure 1:**
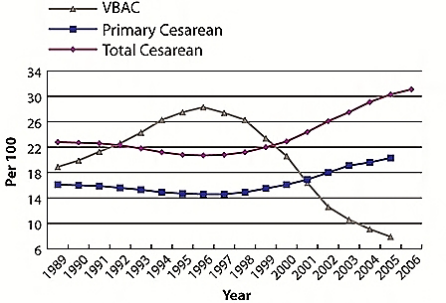
**Total cesarean, primary cesarean, and vaginal birth after cesarean rates, United States, 1983-2006**. (Note: for comparability, 2004 and 2005 primary cesarean and VBAC rates are limited to 37 jurisdictions with unrevised birth certificates, encompassing 69% of 2005 births; 2006 total cesarean rate is preliminary.) Source: U.S. National Center for Health Statistics.

After considering the potential impact of the guidelines on VBAC availability, particularly in community and rural hospitals, ACOG later clarified that the operational definition of "immediately available" was subject to interpretation by the local institution [13]. This clarification did not stop the downward trend, however. In 2001, another influential article appeared in the *New England Journal of Medicine*, which further precipitated the decline in VBAC. Authored by Lydon-Rochelle et al [14], this article reported that the risk of uterine rupture was higher among those women with a previous cesarean whose labor was induced; moreover, induction with prostaglandins conferred a much higher relative risk of uterine rupture. Associated editorials [15] and subsequent media attention following the publication of this article directed the focus toward the rare complication of uterine rupture instead of highlighting the potential benefits of VBAC and the risks of multiple cesareans. As a result, VBAC rates steadily declined over the next decade, to the current rate of 8.2% nationwide [16,17] (Figure 1).

There are no qualitative studies to date that have explored the effect of national practice guidelines from the perspective of obstetricians and midwives in the United States. One study from the United Kingdom, however, qualitatively explored the views of 12 doctors and 13 midwives on decision making around VBAC [18]. Although the question of immediate availability was not raised in this study, the participants thought that guidelines and protocols were not particularly useful due to the contingent nature of decision making. They were also concerned about litigation risk in the event of a poor outcome and suggested that the use of guidelines was not protective in that regard.

There are 2 published US studies which suggest that the ACOG guidelines played an important role in the VBAC decline [16,19]. In a survey of Colorado, Montana, Oregon, and Wisconsin hospitals, Roberts et al [16] found that more than one-third no longer offered VBAC since the issuance of the 1999 ACOG guidelines. Moreover, 68% of the hospitals had changed their policies to require the in-house presence of surgical and anesthesia personnel when a woman desiring VBAC presented in labor [16]. Zweifler and colleagues [19] also discovered a decline in the uptake of a trial of labor in a population-based study in California. The researchers examined birth statistical master files and found that there was a significant decrease in the number of attempted VBACs (from 24% to 13.5%) without corresponding improvement in either neonatal or maternal mortality.

In areas where VBAC is unavailable, women with a previous cesarean are forced to undergo a repeat cesarean or to travel a considerable distance from their homes to find a healthcare provider who will attend them. Some women also opt for a home delivery because they cannot find obstetricians or midwives who can or will attend them in the hospital [16,20,21]. In fact, frustration over a lack of access to VBAC has spawned a grass-roots activist movement to restore the "right" to give birth vaginally after a previous cesarean [20,21]. Concern over these issues prompted a second NIH VBAC Consensus Conference in March 2010 [22]. Although the original intent of the guidelines was to improve patient safety, the evidence presented at this conference suggested that the current policy has not improved outcomes. Women undergoing a repeat cesarean have a significantly increased risk of maternal mortality [23], abnormal placentation in subsequent pregnancies [24], and chronic pain from adhesions later in life [25]. Repeat cesarean is also more costly than VBAC due to increased maternal and neonatal morbidity and increased hospital length of stay compared with VBAC [26]. Although ACOG published a revised set of guidelines following the Consensus Conference, there was essentially no change in the language surrounding immediate availability. The guidelines did state, however, that "patients should be allowed to accept increased levels of risk" if they were clearly informed of the increased risks and management alternatives [[27], p.458].

Given the national mandate to increase access, it is important to explore the factors that either enable providers to offer or constrain them from offering VBAC. The Florida case may be instructive insofar as the state has among the highest cesarean rates (37.2%) and lowest VBAC rates (0.7%) in the United States [28]. The purpose of this qualitative study was to explore providers' perspectives on the effect of the ACOG VBAC guidelines in Florida. The specific aims were to examine the barriers associated with these guidelines, as well as the strategies that providers used to continue to offer VBAC in the current litigious climate.

## Methods

This article is based on a thematic analysis of data from a larger qualitative study of the social, political, and economic factors influencing Florida healthcare providers' decision-making about mode of delivery [29]. The University of Florida Institutional Review Board (IRB) approved the study. Due to the sensitive nature of some of the interview questions, it was essential to be able to assure provider anonymity in order to recruit a sufficient number of participants. To reduce this barrier, a Waiver of Documentation of Informed Consent was obtained during the IRB approval process. At the time of recruitment, participants were given an IRB-approved letter describing the study and their rights as a research subject. They read the letter and verbally agreed to participate, but they were not required to sign a consent form. This facilitated the recruitment process considerably.

### Sample

Three types of providers are licensed to attend deliveries in Florida: obstetricians, certified nurse-midwives (CNMs) and licensed midwives (LMs). Although the majority of CNMs attend hospital births and provide services in collaboration with an obstetrician, LMs usually practice in homes and/or birth centers under a restricted protocol. Obstetricians and most CNMs practice in various hospital settings. These generally include academic medical centers (teaching, or "tertiary, " hospitals with 24-hour obstetrical, anesthesia, and neonatal coverage), or community hospitals, where most providers take call from home. Community hospitals in rural areas often have few obstetricians on staff and no advanced neonatal services. All 3 types of providers are compensated by several forms of payment sources, including private insurance, government-sponsored insurance (Medicaid), or self-pay by the woman and her family.

Obstetricians, CNMs, and LMs in Florida are required by law to demonstrate financial responsibility in the event of a malpractice claim, unless they serve as an officer, employee, or agent of the state or federal government. Those who work in a government capacity have "sovereign immunity" [30] from lawsuits. This means that the state or federal government, rather than the individual practitioner, becomes the defendant in the event of a malpractice suit. Other providers, however, have several options to meet the financial responsibility requirement. According to law, they may obtain professional liability insurance, establish an escrow account, maintain an unexpired, irrevocable letter of credit, or be self-insured under a group trust fund [31].

Several types of providers and healthcare settings were sought in order to explore a broad range of views and services and to help ensure anonymity of the participants. Purposive snowball sampling was used to recruit the obstetricians and midwives. This means that the personal and professional networks of the researchers were used to locate participants. Snowball sampling is particularly effective when trying to access groups that are difficult to recruit [32]. The sampling process began with locating providers known to the author who were likely to be knowledgeable about the subject. These key informants subsequently led to other potential participants. Inclusion criteria were as follows: age of 21 to 70 years, fluent in English, currently licensed to practice in Florida, and willingness to participate in the study. Obstetricians and CNMs had to have current hospital delivery privileges, and LMs were required to be currently providing out-of-hospital birth in either home or birth center settings. Administrators had to meet the general criteria as well as have primary oversight of a licensed, hospital-based labor and delivery unit or freestanding birth center. A total of 24 participants were recruited: 11 obstetricians, 8 CNMs, 4 LMs, and 1 maternity hospital administrator. Characteristics of the sample are shown in Table 1.

**Table 1:** Demographic characteristics of the interview sample (*N *= 24)

	MD *n *= 11	CNM/ARNP^a^ *n *= 9	LM *n *= 4
Age, years			
Mean (*SD*)	46 (12.1)	54 (5.5)	50 (13.2)
Range	30-60	46-61	30-58
Median	49	53	56
Ethnicity, *n *(%)			
Black	1 (4)	0 (0)	0 (0)
Hispanic	0 (0)	1 (4)	0 (0)
White	10 (42)	8 (33)	4 (17)
Gender, *n *(%)			
Female	7 (29)	9 (37)	4 (17)
Male	4 (17)	0 (0)	0 (0)
Type of Hospital, *n *(%)			
Academic Med. Center	2 (8)	0	0
Community	9 (40)	8 (33)	4 (17)
Provides VBAC, *n *(%)			
Yes	9 (38)	2 (8)	1 (4)
No	2 (8)	6 (25)	3 (13)
Years in practice			
Mean (*SD*)	15 (11.3)	19 (9.7)	18 (14.9)
Range	1-31	2-32	5-37
Median	18	21	15
Malpractice suit, *n *(%)			
Yes	8 (33)	3 (13)	1(4)
No	3 (13)	6 (25)^a^	3(13)

Abbreviations: MD, medical doctor; CNM/ARNP, certified nurse-midwife/advanced registered nurse practitioner;

LM, licensed midwife; VBAC, vaginal birth after cesarean.

^a^ARNP administrator (*n *= 1) was included with the CNM group; percentages may not equal 100%.

Interviews were face-to-face, digitally recorded, and lasted from 20 to 50 minutes. They took place in a variety of settings, including provider offices, hospital call rooms, and restaurants. Participants chose the site for the interview, and care was taken to maintain privacy at a level that the participants felt was acceptable. The initial plan was to conduct individual interviews; however, after several partners in the same practice requested to be interviewed together, IRB approval was obtained to conduct either individual or small-group interviews according to the participants' preference. Fourteen participants were interviewed individually: 10 obstetricians, 2 CNMs, 1 LM, and 1 hospital administrator. One midwife group had 2 practice partners, 2 groups had 3 partners, and 1 group consisted of an obstetrician and a CNM. Each group was interviewed together. All of the semi-structured interviews were conducted by the author and were based on an interview guide. The guide included topics such as how women were counselled about mode of delivery, whether the providers tried to influence the woman's decision, and who they thought should be responsible for the decision and why. There were also questions about any restrictions on VBAC in their practice or hospital, including what they told women about VBAC if they did not offer the option. They were also asked to describe how their practice had changed over time in relation to VBAC, whether they had experienced conflict over the issue, and what their thoughts were on out-of-hospital VBAC. Pseudonyms were assigned to protect the participants' identities.

Data collection and analysis was an iterative process. This means that data analysis was a continuing progression, employing themes from early interviews to guide the following themes [32,33]. The interview guide was also modified slightly to further develop emerging themes. Because the providers themselves voiced numerous concerns regarding the influence of the ACOG guidelines and the need to be immediately available, a decision was made early on in the study to explore this aspect in detail. During this process, the research team met periodically to discuss emerging themes and revise coding hierarchies. Memos were used to group themes and to document the analysis process. Participant recruitment ended once thematic saturation was reached, meaning that no new themes were noted in the interview data.

Interviews were transcribed verbatim by the author or by a professional transcriber. NVivo 7.0 software was used for data management throughout the research process. Although it was not possible to validate responses with every participant, themes and representative quotes were reviewed and evaluated for meaning throughout the study by both obstetrician and midwife key informants. In addition, time spent in the field and researcher reflexivity contributed to the validity of the study [32,33].

## Results

Five central themes emerged from the analysis of the interview transcripts: fear of liability, minimizing risk, convenience of cesarean, defining "immediately available, " and marginalization of midwives. Obstetricians tended to be more risk-averse than the midwives, but there were not always clear differences between the groups. Thus, exemplar narratives were selected to demonstrate variations in perspective within a given theme. It is important to note that these differences represent competing ideologies of birth that cannot exclusively be ascribed to a particular provider group.

### Fear of Liability

For a number of providers, fear of liability was a major impediment to offering a trial of labor for women with a previous cesarean. According to Dr. Diane (all names are pseudonyms), who practices in a suburban community hospital, "obstetricians are in a constant *fear *of being sued, so they're taking a path of least resistance." This fear was often generated from experience with a lawsuit. Dr. Arthur, a senior obstetrician in a rural community hospital, offered his perspective:

*If you have a problem *[during a trial of labor], *you are going to get no sympathy from the medico-legal community. They are going to be all over you, and if you end up with a ruptured uterus, you are going to be lucky if you get a viable newborn and you don't have a lot of problems with the mother. And nobody is going to be sympathetic for any unusual pattern on the monitor...you can't tell me of a single failed VBAC that resulted in a ruptured uterus that wasn't a disaster medico-legally*.

Interestingly, fear of liability around VBAC was not limited to those practicing in community and rural hospitals. Even obstetricians in academic medical centers, with 24-hour obstetrical and anesthesia coverage, reported that some of their colleagues refused to allow women a trial of labor at all. When asked why this was so, Dr. David responded: "I think the problem with VBACs is *largely *with lawsuits. I think it's a question of not wanting to get sued!"

Some providers, however, took a more pragmatic approach to their concerns about liability, particularly in terms of how it affected their practice. Dr. Angela put it this way:

*I've had plenty of negative experiences with VBAC. Plenty of people with uterine ruptures, plenty of people with scar dehiscence. Failed VBAC, I had to section them and you're looking through the serosa at the baby! That doesn't mean I'll stop doing it, but it means I do approach it with caution, like I would anything else*.

Midwives were also concerned about potential liability, for both themselves and their obstetrician colleagues. CNM Grace said she thought that obstetricians "very much had to tailor their practice to the legal malpractice climate." Similar to Dr. Angela, however, Grace was not inclined to change her practice based on fear of a lawsuit:

*I just think it's a bunch of crap that you have to change your practice when you know something is safe because somebody might sue you. Anytime you get a less than optimal outcome, people want to blame, people want to sue. You may have done everything right, and it doesn't matter, so you can't live your life being afraid of that...there's so much you *can *control, and things have a way of happening sometimes. It's just kind of a personal philosophy, too. I just think that most long-term midwives get to that point. Otherwise you'd be too afraid to do anything. Birth is amazing, and not always predictable*.

### Minimizing Risk

Of the 11 obstetricians in the study, 4 had sovereign immunity, and 7 reported that they did not carry malpractice insurance due to the high costs of premiums. Data were not collected on the other options for obstetricians. All 12 midwives said they carried malpractice insurance. None had sovereign immunity or the financial means to utilize the other options, such as letters of credit or surety bonds. (Table 1). The providers used several strategies to manage the risks associated with caring for women with a previous cesarean. Avoidance was the most common strategy. According to Dr. Arthur:

*But here at this Level I *[rural, community hospital], *there's no way that they can meet the requirements of ACOG's recommendation. So I think there are too many areas that you can't cover trying to do a VBAC, and as a result of that, I have chosen to just deliver all of my previous sections by cesarean section*.

Other providers in community hospitals continued to offer a trial of labor, but they also attempted to minimize their risks of liability by recommending it only to the most motivated and adamant women. Dr. Patricia did not feel comfortable encouraging women toward VBAC unless they expressed a "strong" desire to try it:

*They really do need to express an interest in it. I do feel really hampered by being in the State of Florida with no professional liability insurance. So the safest thing for the *baby *is a repeat C-section. I will never get hammered on that in the court of law. I will get hammered in a court of law allowing a VBAC to occur*.

Midwives who were delivering babies without an in-house consultant physician or anesthetist felt particularly vulnerable. CNM Stacy, a community-based midwife in private practice, expressed relief about no longer attending VBACs because of her experiences with delays during emergencies:

*I was somewhat saddened when we stopped doing them because I think in our practice we had a very good success rate for delivering previous C-sections. But one time it took over 15 minutes for somebody from anesthesia to get there, and when you're sitting there and the baby is going bad, it was a difficult position to be in. So I finally made peace with it*.

### Convenience of Cesarean

Both obstetricians and midwives said that the convenience of scheduling a repeat cesarean was appealing for several reasons. First, many women preferred to avoid labor and appreciated the convenience and control that repeat cesarean afforded. Second, having to remain immediately available throughout a trial of labor imposed significant lifestyle and practice limitations, particularly for providers in rural, solo, or small-group community practices. Dr. Charles, a community obstetrician in practice with a midwife, offered his thoughts on why the convenience of repeat cesarean was attractive for obstetricians:

*It's certainly easier to do a repeat C-section, so why not just say, 'Shoot, I don't have to deal with VBACs, great! The few patients that want to go out of town *[for a VBAC] *can go there, and I get to have a little bit easier life.' I think when you get to the heart of it, that's what's going on*.

Some of the midwives were critical of obstetricians, however, for what they viewed as a promotion of repeat cesarean for their own convenience. LM Rosa offered a typical perspective:

*I have been appalled at how many OBs *[obstetricians] *will let them pick the date on their *first OB visit *for their repeat cesarean. Repeat cesareans are not only OK here, they're promoted! They can pick the date, which is very convenient...and they're selling, they're *selling cesareans.

There were some obstetricians, however, who felt that women's choices took precedence over the doctor's convenience. According to Dr. Hanna, a community obstetrician in a small group practice, "it's much easier for us to schedule a C-section, but if it's [VBAC] something that the patient wants, then we certainly give them that opportunity."

### Defining "Immediately Available"

Regardless of size or location, all of the hospitals in the sample utilized the ACOG guidelines as the defining standard of care for VBAC. Definitions of "immediately available, " however, varied considerably from hospital to hospital. According to Dr. Fay, who worked in a mid-size, urban community hospital:

*Immediately available in the hospital's definition is within 10 minutes from the unit *[labor and delivery]*. Our office is 3 blocks away, my house is within the 10-minute window. Unless there's a problem, I am basically doing what I would normally do on call, which is not to be more than 10 minutes away from the hospital, anyway. It really doesn't change the time factor*.

Dr. Patricia, who practiced in a mid-size, suburban community hospital, stated:

*We require ourselves to be in-house. We have a very strange rule here that does not exist in any other hospital...if we have Pitocin, an epidural, or a VBAC in labor, the provider *has *to be in the hospital with the patient. We cannot leave the facility. There's no perineal obstetrics. We are here*.

In other community hospitals, the immediate availability of an anesthetist was the central issue. However, as Dr. Megan described, the decision about whether or not to allow VBACs depended heavily on the political power of both the anesthesia and obstetrical groups at the Medical Staff meetings:

*Our issue has been that our anesthesia group does not have a dedicated anesthesia provider for L&D *[labor and delivery]*. There were also some obstetrics groups that also supported that--they weren't offering VBAC and didn't have any desire to consider offering that service. So current hospital policy is that we're not able to offer a VBAC*.

### Marginalization of Midwives

Interestingly, there is no mention of the role of midwives in the 2010 ACOG VBAC guidelines. Because the recommendation is for the immediate availability of an *obstetrician and anesthesiologist*, the midwives in this study felt they were marginalized in terms of care provision and excluded from the policy-making process. There were various reasons, they thought, for why this had occurred. According to CNM Katherine:

*We were doing VBACs with no problem in the hospital, and then, the doctors dropped their malpractice insurance, and we weren't able to do VBACs, even with the doctors there. Even if the woman wanted the midwives to be giving the care*.

Since the physicians were required by the hospital to be immediately available during the labor and birth, some of the midwives in private practice discovered that they were being excluded for financial reasons as well. According to Florida billing practices, only 1 provider can be paid for the delivery. In most cases, the midwives found that their consultant physicians were opting to conduct the births themselves and collect payment. CNM Barbara stated:

*Then the ACOG shift happened where they *[hospital policy-makers] *decided the OB had to be in-house, and he *[the obstetrician] *decided he's not going to be there in the house and not get the money for the birth. So we had to stop doing them *[VBACs].

Some obstetricians thought that restricting midwives from the care of women with a previous cesarean was an unwise strategy, however. Dr. Charles, who practiced in a community hospital with a midwife, described his perspective on collaborative practice arrangements:

*Now I allow my midwife to take care of VBACs. Once the patient was in active labor, I was always within 10 minutes away, and I was always in the hospital for the delivery, no matter what! Now the other group won't allow their midwives to take care of a VBAC patient, which I think is *stupid*, because, if anything, the VBAC patient needs more one-on-one kind of coaching and encouragement, and the midwife's in a position to do that. I think our VBAC success was as good if not better with the midwife doing it. And we had a team...our system was such that they had *no *financial disincentives to call me. But they never called me unless it was appropriate, so it worked out fine*.

Most LMs in Florida are self-employed and have small home-birth or birth center practices. Although they are not required to have a collaborating physician of record to practice, they are restricted by the rules associated with their practice act when caring for a woman with a previous cesarean. LM Sylvia stated:

*We *have *to have them signed up by an obstetrician with hospital privileges as likely to have a normal labor and birth. We may not even do prenatal care on somebody in that situation without having a signed collaborative management agreement. Our back-up physician, as well as an anesthesiologist, is required by the hospital to be present the whole time a VBAC is in labor, and so he's not able to make that time commitment. So he's not doing VBACs; thus, he's not signing us off for doing VBACs*.

Not all LMs in Florida are in this predicament, however. Some are still able to find collaborating physicians to sign them off for a home birth, although attempting a trial of labor in a birth center is no longer allowed [34]. Thus, birth centers in Florida are unable to offer VBAC legally, and midwives are concerned that women's choices are declining as a result. According to LM Jennifer:

*I would say we've been getting between 6 and 12 inquiries a month *[about VBAC]*. And that's not women who are choosing out-of-hospital birth as a priority. They have gone ahead and called a bunch of OBs and hospitals and realized the fact that their choice is diminished...it's heartbreaking*.

## Discussion

In their summary statement, the NIH VBAC Consensus Conference Panel put forth the recommendation to reassess the requirement for immediate availability in community hospitals. Specifically, they urged providers, hospitals, professional liability insurers, and consumers to work together to try to mitigate the "chilling effect" of the current medical-legal climate on VBAC access [22]. It is interesting to note, however, that VBAC guidelines from other developed countries, such as the United Kingdom [35,36] and New Zealand [37], also recommend hospital delivery with immediate cesarean section capabilities for women with a previous cesarean. For example, the Royal College of Obstetricians and Gynaecologists' Green-Top Guidelines [35] state that "planned VBAC should be attempted in a suitably staffed and equipped delivery suite, with continuous intrapartum care and monitoring and available resources for immediate cesarean section and advanced neonatal resuscitation" [p. 9]. Although this recommendation is based primarily on consensus and expert opinion rather than empirical evidence [38], it is clear that there *is *a level of international consensus that VBAC requires close vigilance during labor. Unfortunately, however, the recommended guidelines do not provide solutions to the real-world problem of staffing obstetrical units to meet the needs of women with a previous cesarean. How, then, can change be accomplished, particularly in the privatized, for-profit environment of US healthcare?

As the NIH VBAC Consensus Conference Panel suggested, change in the United States will require the input and cooperation of a number of various groups in order to increase women's access. Given that VBAC rates are higher in the integrated and socialized healthcare systems of countries such as the Netherlands and New Zealand, it is apparent that system organization is part of the problem. The narratives of the providers in this study provide some nuanced examples of what works and what does not work in relation to offering VBAC as an option. For example, having a clear definition of "immediately available" as hospital policy allowed 1 group of obstetricians to continue to offer VBAC in a community hospital. Another group was able to maintain the option of VBAC by committing to remain in-house during a trial of labor. In yet another example, cooperation between a midwife and an obstetrician without financial motive facilitated a successful "team" approach which maximized VBAC success.

Given the experiences of other providers in the study, it is reasonable to expect that VBAC cannot be offered in every hospital with obstetrical services. Fear of liability is a particularly potent issue. Research from other developed countries [2,18,38-41] indicates that malpractice concerns around VBAC are not confined to the United States. Moreover, other obstetrical emergencies, such as prolapsed cord, occur with frequency similar to uterine rupture with VBAC, yet there is no mandate for an obstetrician to be immediately available for those rare situations [22]. Clearly, the emphasis on legal risk with VBAC is out of proportion to the available evidence regarding safe practice [42]. As several of our participants have pointed out, practice should be based on evidence and good judgment rather than fear of liability. Perhaps one way to accomplish this in a country as large and diverse as the United States is to work at regional and state levels to develop risk stratification and appropriate referral systems so providers have options other than choosing to "just deliver all of my previous sections by cesarean section." The work of the Northern New England Perinatal Quality Improvement Network is an example of such an initiative and is discussed in detail on their website [43].

The marginalization of midwives in the State of Florida and in the United States is another key factor in the uptake of VBAC. Notably, midwives are not even *mentioned *in any version of the ACOG guidelines; however, they are included as part of a team approach to caring for women with a previous cesarean in guidelines from both the United Kingdom [35,36] and New Zealand [37]. In this study, the midwives had little control about whether or not they were able to participate in VBACs. Much of their ability to do so was dependent on the setting and/or the willingness of the obstetricians they worked with. They were also frustrated by the lack of proactive solutions to the limited access to VBAC in Florida. Many women with a previous cesarean were seeking their care in hopes of having a vaginal delivery. Some of these women were traumatized over their previous cesarean and desired the continuous presence of a supportive caregiver during labor in order to achieve a vaginal birth. Yet, the midwives felt that "their hands were tied" in terms of being able to be part of the solution. The midwives' narratives also provided some important information about the increasing number of requests for out-of-hospital VBAC in Florida. Although the number of women involved is small, MacDorman et al [1] point out that this is an increasing trend in the United States in response to limited VBAC access.

There are limitations to this study. This small sample from 1 southeastern state may not reflect the situation in other states or regions, which could have different problems with access, provider relationships, or medical-legal climate. Additionally, most of the interview data came from white, female providers. Although this is representative of Florida's provider profiles for both obstetricians and midwives, it fails to capture the perspectives of minority providers.

This study also has a number of strengths. It is the first known study to explore the effects of the ACOG Guidelines from the perspective of maternity care providers. The narratives here provide insight into how some obstetricians continue to offer VBAC in community hospitals and why others do not. The data also illuminate some of the reasons for the small but steady increase in out-of-hospital VBACs in Florida from the perspective of midwives.

According to ACOG [3], the intent of the guidelines was to improve patient safety, not to decrease access to VBAC. As this small study reveals, however, the barriers for providers are substantial. Clearly, more research is needed on the impact of practice guidelines on providers as well as the women they serve.

## Conclusions

Although access to VBAC remains very limited in Florida, there has been little effort to date by leaders in obstetrics or public health to address the issue. The recommendations from the NIH VBAC Consensus Conference [2] urged ACOG to reconsider the requirement for immediately availability in order to increase women's access. Although ACOG responded with a liberalized set of guidelines and encouraged obstetricians to consider women's autonomy, there was virtually no change to the immediately available requirement. In light of what the providers in this study have said about the ubiquitous nature of this policy, it seems unlikely that allowing patients "to accept increased levels of risk" [3] will invoke much change in access to VBAC in Florida without tort reform and a cooperative statewide effort.

## Competing interests

The author declares that they have no competing interests.

## Authors' contributions

The author was primarily responsible for the conception and design of the study, as well as the drafting of the manuscript. This research was conducted as part of the requirements for a doctoral degree in nursing.

## Pre-publication history

The pre-publication history for this paper can be accessed here:

http://www.biomedcentral.com/1471-2393/11/72/prepub

## References

[B1] MacDorman M Declercq E Menacker F Recent trends and patterns in cesarean and vaginal birth after cesarean (VBAC) deliveries in the United States Clin Perinatol 2011 38 2 179 192 10.1016/j.clp.2011.03.007 21645788

[B2] Homer CSE Johnston R Foureur MJ Birth after caesarean section: changes over a nine-year period in one Australian state Midwifery 2011 27 2 165 169 10.1016/j.midw.2009.04.009 19773099

[B3] Cragin EB Conservatism in obstetrics NY Med J 1916 104 1 3

[B4] Bonanno C Clausing M Berkowitz R VBAC: a medicolegal perspective Clin Perinatol 2011 38 2 217 225 10.1016/j.clp.2011.03.005 21645790

[B5] National Institutes of Health. Cesarean childbirth. Washington, DC National Institutes of Health, 1981 NIH publication no. 82-2067

[B6] Flamm BL Lim OW Jones C Fallon D Newman LA Mantis JK Vaginal birth after cesarean section: results of a multicenter study Am J Obstet Gynecol 1988 158 5 1079 1084 3369487 10.1016/0002-9378(88)90224-4

[B7] Flamm BL Goings JR Liu Y Wolde-Tsadik G Elective repeat cesarean delivery versus trial of labor: a prospective multicenter study Obstet Gynecol 1994 83 6 927 932 10.1097/00006250-199406000-00005 8190433

[B8] MacDorman MF Menacker F Declercq E Cesarean birth in the United States: epidemiology, trends, and outcomes Clin Perinatol 2008 35 2 293 307 v 10.1016/j.clp.2008.03.007 18456070

[B9] ACOG practice bulletin. Vaginal birth after previous cesarean delivery. Number 5, July 1999 (replaces practice bulletin number 2, October 1998). Clinical management guidelines for obstetrician-gynecologists. American College of Obstetricians and Gynecologists Int J Gynecol Obstet 1999 66 197 204 10468354

[B10] ACOG Committee Opinion No. 64: guidelines for vaginal delivery after a cesarean birth 1988 American College of Obstetricians and Gynecologists. Washington, DC

[B11] Harer WB Jr Vaginal birth after cesarean delivery: current status JAMA 2002 287 20 2627 2630 10.1001/jama.287.20.2627 12020285

[B12] McMahon MJ Luther ER Bowes WA Jr Olshan AF Comparison of a trial of labor with an elective second cesarean section N Engl J Med 1996 335 10 689 695 10.1056/NEJM199609053351001 8703167

[B13] Zinberg S Cesarean delivery resources need to be available during VBAC trial of labor ACOG Today 1999 43 6 2

[B14] Lydon-Rochelle M Holt VL Easterling TR Martin DP Risk of uterine rupture during labor among women with a prior cesarean delivery N Engl J Med 2001 345 1 3 8 10.1056/NEJM200107053450101 11439945

[B15] Greene MF Vaginal delivery after cesarean section--is the risk acceptable? N Engl J Med 2001 345 1 54 55 10.1056/NEJM200107053450108 11439949

[B16] Roberts RG Deutchman M King VJ Fryer GE Miyoshi TJ Changing policies on vaginal birth after cesarean: impact on access Birth 2007 34 4 316 322 10.1111/j.1523-536X.2007.00190.x 18021147

[B17] Russo C Wier L Steiner C Hospitalizations related to childbirth, 2006. (HCUP Statistical Brief #71) http://www.hcup-us.ahrq.gov/reports/statbriefs/sb71.pdf 21510028

[B18] Kamal P Dixon-Woods M Kurinczuk JJ Oppenheimer C Squire P Waugh J Factors influencing repeat caesarean section: qualitative exploratory study of obstetricians' and midwives' accounts BJOG 2005 112 8 1054 1060 10.1111/j.1471-0528.2005.00647.x 16045517

[B19] Zweifler J Garza A Hughes S Stanich MA Hierholzer A Lau M Vaginal birth after cesarean in California: before and after a change in guidelines Ann Fam Med 2006 4 3 228 234 10.1370/afm.544 16735524 PMC1479438

[B20] ICAN Online http://www.ican-online.org/

[B21] Rubin R Mother's stories NIH Consensus Development Conference on Vaginal Birth After Cesarean: New Insights 2010 Bethesda, MD

[B22] Cunningham F Bangiwala S Brown S Dean T Frederiksen M Rowland Houge C King T Spencer Lukacz E McCullough L Nicholson W Petit N Probstfield JL Viguera AC Wong CA Zimmet SC National Institutes of Health Consensus Development Conference Statement: Vaginal Birth after Cesarean: New Insights Obstet Gynecol 2010 115 6 1279 1295 10.1097/AOG.0b013e3181e459e5 20502301

[B23] Guise JM Denman MA Emeis C Marshall N Walker M Fu R Janik R Nygren P Eden KB McDonagh M Vaginal birth after cesarean: new insights on maternal and neonatal outcomes Obstet Gynecol 2010 115 6 1267 1278 10.1097/AOG.0b013e3181df925f 20502300

[B24] Silver RM Landon MB Rouse DJ Leveno KJ Spong CY Thom EA Moawad AH Caritis SN Harper M Wapner RJ Sorokin Y Miodovnik M Carpenter M Peaceman AM O'Sullivan MJ Sibai B Langer O Thorp JM Ramin SM Mercer BM National Institute of Child Health and Human Development Maternal-Fetal Medicine Units Network Maternal morbidity associated with multiple repeat cesarean deliveries Obstet Gynecol 2006 107 6 1226 1232 10.1097/01.AOG.0000219750.79480.84 16738145

[B25] Nisenblat V Barak S Griness OB Degani S Ohel G Gonen R Maternal complications associated with multiple cesarean deliveries Obstet Gynecol 2006 108 1 21 26 10.1097/01.AOG.0000222380.11069.11 16816051

[B26] DiMaio H Edwards RK Euliano TY Treloar RW Cruz AC Vaginal birth after cesarean delivery: an historic cohort cost analysis Am J Obstet Gynecol 2002 186 5 890 892 10.1067/mob.2002.123073 12015504

[B27] ACOG Practice Bulletin no. 115: vaginal birth after previous cesarean delivery Obstet Gynecol 2010 116 2 Pt 1 450 463 20664418 10.1097/AOG.0b013e3181eeb251

[B28] Hamilton BE Martin JA Ventura SJ Births: preliminary data for 2008 NVSR 2010 57 16 1 23 25073731

[B29] Cox KJ Previous cesarean in Florida: healthcare providers' decision-making about mode of delivery Doctoral Dissertation 2009 University of Florida, College of Nursing

[B30] The 2011 Florida Statutes Health Professions & Occupations: General Provisions, Title 32, § 456.048 (1)(2a) http://www.leg.state.fl.us/Statutes/index.cfm?App_mode=Display_Statute&Search_String=&URL=0400-0499/0456/Sections/0456.048.html

[B31] The 2011 Florida Statutes Medical Practice Act, Title 32, § 458.320 http://www.leg.state.fl.us/statutes/index.cfm?mode=View%20Statutes&SubMenu=1&App_mode = Display_Statute&Search_String = financial+responsibility&URL = 0400-0499/0458/Sections/0458.320.html

[B32] Patton M Qualitative Research & Evaluation Methods 2002 3 Thousand Oaks, CA: Sage

[B33] Richards L Morse J Readme First for a User's Guide to Qualitative Methods 2006 2 Thousand Oaks, CA: Sage

[B34] The 2011 Florida Statutes 2011 Birthing Center Standards, Rule 59A-11 https://www.flrules.org/gateway/RuleNo.asp?title=BIRTH%20CENTER%20STANDARDS%20AND%20LICENSURE&ID=59A-11.009

[B35] Royal College of Obstetricians and Gynaecologists Birth after previous caesarean birth (Green-top guideline 45) http://www.rcog.org.uk/womens-health/clinical-guidance/birth-after-previous-caesarean-birth-green-top-45

[B36] National Collaborating Centre for Women's and Children's Health Caesarean Section 2004 London: RCOG Press

[B37] Group NZG Care of Women with Breech Presentation or Previous Caesarean Birth 2004 Wellington: New Zealand Guidelines Group

[B38] Foureur M Ryan CL Nicholl M Homer C Inconsistent evidence: analysis of six national guidelines for vaginal birth after cesarean section Birth 2010 37 1 3 10 10.1111/j.1523-536X.2009.00372.x 20402716

[B39] Bryant J Porter M Tracy S Sullivan E Caesarean birth: consumption, safety, order, and good mothering Soc Sci Med 2007 65 6 1192 1201 10.1016/j.socscimed.2007.05.025 17590252

[B40] Gunnervik C Josefsson A Sydsjö A Sydsjö G Attitudes towards mode of birth among Swedish midwives Midwifery 2010 26 1 38 44 10.1016/j.midw.2008.04.006 18632194

[B41] Vimercati A Greco P Kardashi A Rossi C Loizzi V Scioscia M Loverro G Choice of cesarean section and perception of legal pressure J Perinat Med 2000 28 2 111 117 10.1515/JPM.2000.014 10875095

[B42] Albers LL Safety of VBACs in birth centers: choices and risks Birth 2005 32 3 229 231 10.1111/j.0730-7659.2005.00375.x 16128979

[B43] VBAC Project http://www.nnepqin.org/site/page/vbac

